# Therapeutic Role of Hematopoietic Stem Cells in Autism Spectrum Disorder-Related Inflammation

**DOI:** 10.3389/fimmu.2013.00140

**Published:** 2013-06-10

**Authors:** Dario Siniscalco, James Jeffrey Bradstreet, Nicola Antonucci

**Affiliations:** ^1^Department of Experimental Medicine, Second University of Naples, Naples, Italy; ^2^Centre for Autism – La Forza del Silenzio, Caserta, Italy; ^3^Cancellautismo, Florence, Italy; ^4^International Child Development Resource Center, Cumming, GA, USA; ^5^Biomedical Centre for Autism Research and Treatment, Bari, Italy

**Keywords:** autism, hematopoietic stem cells, cell transplantation, cytokines, inflammation

## Abstract

Autism and autism spectrum disorders (ASDs) are heterogeneous, severe neuro-developmental disorders with core symptoms of dysfunctions in social interactions and communication skills, restricted interests, repetitive – stereotypic verbal and non-verbal behaviors. Biomolecular evidence points to complex gene-environmental interactions in ASDs. Several biochemical processes are associated with ASDs: oxidative stress (including endoplasmic reticulum stress), decreased methylation capacity, limited production of glutathione; mitochondrial dysfunction, intestinal dysbiosis, increased toxic metal burden, and various immune abnormalities. The known immunological disorders include: T-lymphocyte populations and function, gene expression changes in monocytes, several autoimmune-related findings, high levels of *N*-acetylgalactosaminidase (which precludes macrophage activation), and primary immune deficiencies. These immunological observations may result in minicolumn structural changes in the brain, as well as, abnormal immune mediation of synaptic functions. Equally, these immune dysregulations serve as the rationale for immune-directed interventions such as hematopoietic stem cells (HSCs), which are pivotal in controlling chronic inflammation and in the restoration of immunological balance. These properties make them intriguing potential agents for ASD treatments. This prospective review will focus on the current state-of-the-art knowledge and challenges intrinsic in the application of HSCs for ASD-related immunological disorders.

## Autism Spectrum Disorders: Overview

Autism and autism spectrum disorders (ASDs) are severe heterogeneous neuro-developmental abnormalities characterized by dysfunctions in social interactions and communication skills, restricted interests, repetitive, and stereotypic verbal and non-verbal behaviors (American Psychiatric Association, 2000; Stankovic et al., 2012). Autism related disorders are increasing at an alarming rate and have now affected 2% (3.23% of boys) of US school-aged children (Blumberg et al., 2013).

Autism spectrum disorders have multifactorial and polygenic features which include: a complex combination of genetic, epigenetic, and environmental interactions (i.e., infectious agents, air pollution, organophosphates, heavy metals) (Herbert, 2010; Toro et al., 2010). An early inflammatory process has been proposed as the potential etiology of ASDs (Depino, 2013). This hypothesis is supported by animal models and has been extensively reviewed by Meyer et al. (2009). Within that review, the authors refer to the long-term consequences of prenatal immune provocation in the rodent-model (Meyer et al., 2006). Specifically, experiments exposed pregnant mouse dames to PolyI:C (a synthetic chemical resembling viral RNA) via tail vein infusion at either mid or late gestation. Rapid induction of CNS cytokine activity was noted in the pups which coincided with reduction in reelin production. This effect was noted to have lifelong consequences on both the structure and immune status of the CNS in the mice following *in utero* exposure to a viral analog. Potentially related to these animal models are the recent observations of elevated *N*-acetylgalactosaminidase (Nagalase) levels in the blood of children with ASDs (Bradstreet et al., 2012). This may reflect ongoing viral latency, since Nagalase is observed to be elevated in numerous viral-mediated acute and chronic disease states. This latency may have been present from *in utero* or the early postnatal period.

Others have observed minicolumn abnormalities in ASDs (Casanova et al., 2006). As recently reviewed by Folsom and Fatemi (2013), reelin is integrally involved in ASD pathophysiology and is further a regulator of minicolumn structure and function. It therefore appears likely that in many ASDs an early (*in utero* or early postnatal period) immunological insult disrupts reelin signaling and cytokine communication in the CNS.

Still other biochemical and cellular processes are reportedly associated with ASDs: oxidative stress, endoplasmic reticulum stress, decreased methylation capacity, limited production of glutathione, mitochondrial dysfunction, intestinal dysbiosis, increased toxic metal burden, immune dysregulation, immune activation of neuroglial cells (Siniscalco et al., 2012a). These findings may represent consequences of the primary etiological processes. Regardless of the cause or effect nature of these observed abnormalities, it is probably that these issues interfere with proper CNS functioning in ASDs, and as such they are reasonable targets for therapeutic interventions.

There is, however, a lack of consensus regarding the etiopathologies of ASDs (Siniscalco et al., 2012a). Current medication usage attempts to reduce the problematic behaviors, without addressing the basic underlying etiologies (Chadman et al., 2012; Hampson et al., 2012). These medications often lack evidence of safety and efficacy for the core features of ASD, and instead target maladaptive behaviors and comorbid psychopathology (i.e., irritability, depression, anxiety, hyperactivity, and obsessive-compulsive behaviors) (Siegel and Beaulieu, 2012).

Concerns over safety and limited availability of approved psychotropic medications for children in general, has been encouraging the development of biomedical treatments to target specific biological issues or symptoms. These include the use of: melatonin, acetylcholinesterase inhibitors, naltrexone, carnitine, tetrahydrobiopterin, vitamin C, glutamate antagonists, special dietary supplements, hyperbaric oxygen treatment, immunomodulation and anti-inflammatory treatments, oxytocin, acupuncture, music therapy, and vision therapy (Filipek et al., 2006; Rossignol, 2009; Bradstreet et al., 2010; Wong and Sun, 2010). Several behavioral options are also currently used as effective intervention strategy for autism (Kasari and Lawton, 2010; Vismara and Rogers, 2010).

In the midst of these various therapies, stem cell therapies are emerging as the future of molecular and regenerative medicine (Siniscalco et al., 2012b), and they are providing new opportunities for ASD interventions (Siniscalco et al., 2012a; Siniscalco, 2013). Novel findings on the molecular and cellular basis of ASDs indicate that at least some features of ASDs may be amenable to stem cell therapy (Siniscalco, 2012).

## Autism Spectrum Disorders: CNS Inflammatory Conditions

One difficult feature of the immune dysregulation in ASDs appears to be abnormal regulation of the blood brain barrier (BBB) (Theoharides and Zhang, 2011). The BBB functions are complex and incompletely understood, however, it is clear that the BBB both produces and regulates cytokines, and serves as an immunological interface between the CNS and the peripheral immune system (Siegel and Zalcman, 2008). Hematopoietic stem cells (HSCs) may represent one of the few effective interventions to restore proper regulation of the BBB in ASDs. Stem cells have been recently proposed as elective candidate for modeling BBB (Lippmann et al., 2013). A large number of endogenous HSCs was found in brain (Bartlett, 1982). These HSCs provide constant generation of macrophagic cells without the disturbance of BBB. Macrophagic cells contribute to the normal homeostasis of brain function by removing cellular debris, such as myelin fragments.

Exogenous transplanted stem cells are able to migrate into CNS and retain the differentiation capacity (Simard and Rivest, 2004). Clearly, the BBB allows the passage of stem cells from the blood into the brain or the spinal cord (de Munter and Wolters, 2013), where they can exert their roles. Interestingly, it has been demonstrated that stem cells, *in vitro* differentiated in epithelial cells, possess many BBB-related attributes, such as well-organized tight junctions, expression of nutrient transporters, and polarized efflux transporter activity (Lippmann et al., 2012). These properties are very useful in restoring BBB disruption. In this way, in ASDs, transplanted stem cells could restore the BBB characteristics.

Accumulating evidence points to a chronic up-regulation of inflammatory cytokines in the ASD brain (Ginsberg et al., 2012; Rose et al., 2012). Recently, a role of neuroinflammation and apoptosis mechanisms in the etiology of autism has been proposed (El-Ansary and Al-Ayadhi, 2012), as several biochemical parameters related to inflammation were found up-regulated in children with ASDs (El-Ansary and Al-Ayadhi, 2012; Siniscalco et al., 2012c). Chronic peripheral and central alterations in the inflammatory response have been reported in ASDs (Depino, 2013). Neuroinflammatory evidence was further documented by remarkably elevated levels of cerebrospinal fluid tumor necrosis factor-alpha (TNF-α) in ASDs (Chez et al., 2007). TNF-α profoundly inhibits synaptic communication (Zhang and Dougherty, 2011). Correlations between pro-inflammatory cytokine levels and autistic symptoms have been reported (Buehler, 2011). Interestingly, the cerebellum and temporal cortex of autistic children show decreased glutathione (GSH/GSSG) redox/anti-oxidant capacity and increased oxidative stress associated to a chronic inflammatory response (Rose et al., 2012). Glutathione is a critical intracellular anti-oxidant and children with ASDs have documented deficiencies in the reduced form (James et al., 2006). Lowered GSH levels indicate an adaptive response to ongoing inflammation or infection. In addition, it has been proposed that hyperbaric oxygen therapy may mediate the noted benefits in ASDs via an anti-inflammatory response, perhaps through mobilization of anti-inflammatory CD34+ stem cells from the bone marrow (Rossignol et al., 2012).

## Pro-Inflammatory Indications from Blood Studies in ASDs

At a molecular level, the transcriptional factor, nuclear factor-κB (NF-κB) DNA binding activity was found elevated in peripheral blood samples of children with autism (Naik et al., 2011). NF-κB is an important gene involved in the mediation of inflammation and apoptosis, indicating that pro-inflammatory processes in autism could be up-regulated by this transcriptional factor. Increased NF-κB expression levels were found also in post-mortem samples of orbito-frontal cortex from autistic patients, further indicating a neuro-inflammatory condition (Young et al., 2011). Peripheral blood mononuclear cells (PBMCs) demonstrate significant aberrations in autistic children (Siniscalco et al., 2012c). In autism, these cells are committed to a pro-inflammatory state, via significantly more pro-inflammatory cytokines, including TNFα and several interleukins, IL-1ßand IL-6. The ratio of pro-inflammatory/anti-inflammatory cytokines, TNFα/tumor necrosis factor receptor II (TNFR-II) was also higher in autistic patients (Jyonouchi et al., 2001). The pro-inflammatory cytokine IL-6 was found increased also in the cerebellum of autistic children (Wei et al., 2011).

## Gastrointestinal Immune Alterations in ASDs

In addition to the reported CNS immune issues in ASDs, the intestinal tract (GI) has been studied as a source of chronic inflammation in this population. Recently, extensive evaluation of GI transcriptomes of a group of children with ASDs and GI symptoms were compared to both health tissue, as well as known cases of Crohn’s disease (CD) and ulcerative colitis (UC) (Walker et al., 2013). Significant inflammatory transcriptome overlap was observed between the ASD/GI group and both CD and UC. However, the ASD/GI group demonstrated some distinctions in the inflammatory pattern. Various stem cell therapies have been proposed for CD and UC, which may have application to ASD/GI issues as well, but this is a complex issue. One group recently demonstrated dysregulation of immune-hematopoiesis in colitis mediated by inflammatory cytokines, with increased proliferating HSCs in the bone marrow and spleen. They further demonstrated increased granulocyte-monocyte progenitor (GMP) production at the expense of erythroid and lymphoid progenitors. GMPs were shown to exacerbate the colitis. The Autologous Stem Cell Transplantation International Crohn’s Disease (ASTIC) trial recently published the first round of data (Hawkey, 2012). Bone marrow derived and expanded CD34+ stem cells were infused intravenously with some significant positive results (the Crohn’s Disease Activity Index (CDAI) fell from 324 (median, interquartile range 229–411) to 161 (85–257, *n* = 17).

## Hematopoietic Stem Cells and the Inflammatory State in Autism

Hematopoietic stem cells take a pivotal role in controlling chronic inflammation and in creating immune regulation, and are the cells responsible for forming blood and immune cells. They were found in circulating blood, the spleen, and bone marrow and are characterized by specific cell markers belonging to the cluster of differentiation family (CD34, CD59, CD90, and CD117). HSCs show self-renewal, mobilization, and multipotent differentiation capacities, as they are able to give rise to the myeloid (including monocytes and macrophages) and lymphoid cells, replenishing all blood cell types, and providing homeostasis of blood cells (Sieburg et al., 2011). HSCs are also able to home to the damaged sites (Kavanagh and Kalia, 2011). HSC transplantation has gained much consideration as therapy for hematological malignancies and for the treatment of severe autoimmune diseases (Muraro and Uccelli, 2010).

Interestingly, it has been demonstrated that HSCs are strictly connected with inflammation. Several inflammatory signaling molecules are essential to the HSC response (Baldridge et al., 2011; Boiko and Borghesi, 2012), indicating that the local microenvironment plays an instructive role in stem cell fate (Smith and Calvi, 2013). While in normal condition HSCs travel through peripheral blood at low number, they are strongly mobilized by inflammation (Wright et al., 2001). The activation of inflammatory cytokine signaling pathways promotes transcriptional changes to drive immune and plasticity responses in HSCs (Baldridge et al., 2011). Indeed, in inflammation processes, increased levels of cytokines and hematopoietic growth factors trigger mobilization and proliferation of HSCs, driving a quantitative *in vivo* expansion of the hematopoietic tissue (Möhle and Kanz, 2007). HSCs have demonstrated powerful effects also on acute inflammation.

The ability of HSCs to traffic to sites of inflammation outside of the bone marrow (Granick et al., 2012) suggests that they could be a useful tool in treating inflammatory processes related to autism pathology.

Pro-inflammatory molecules released in ASDs could be able to recruit HSCs to the sites of major inflammation processes, where these cells could exert their beneficial actions against inflammation.

**Figure 1:**
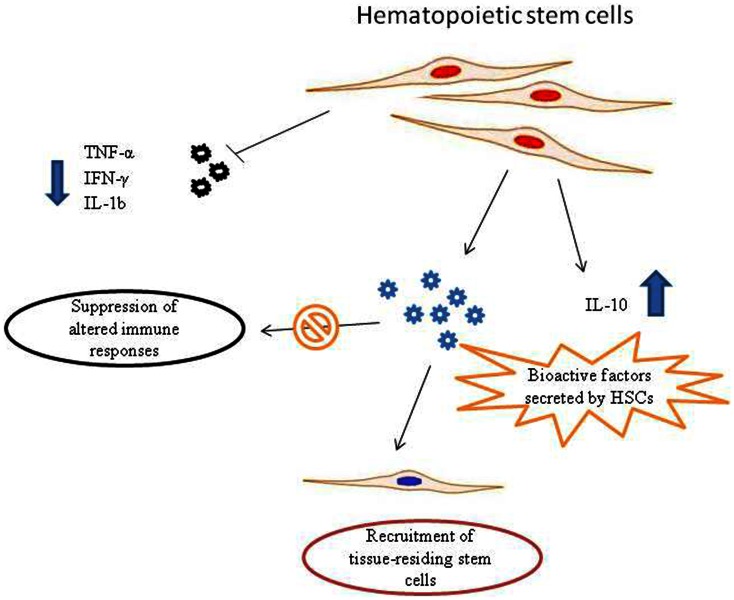
**Possible mechanisms of action of hematopoietic stem cells (HSCs) in autism spectrum disorder (ASD) therapy**. Pro-inflammatory molecules released in ASDs could recruit HSCs to the site of inflammation. HSCs synthesize and release a broad variety of biofactors, including cytokines, chemokines, and growth factors. These bioactive molecules secreted from stem cells are able to suppress aberrant immune responses and stimulate recruitment, retention, and activation of tissue-residing stem cells. In addition, HSCs are able to down-regulate the pro-inflammatory TNF-α, IFN-γ, and IL-1, that are responsible for neuroinflammatory processes in ASDs, as well as to up-regulate the anti-inflammatory cytokine IL-10.

What are the exact molecular mechanism of action are still to be fully elucidated. However, HSCs show paracrine ability (Rosenberg et al., 2012). As other stem cell subtypes, HSCs are able to synthesize and release a broad variety of cytokines, chemokines, and growth factors. These bioactive factors secreted from stem cells suppress the altered immune responses, inhibit apoptosis and stimulate recruitment, retention, mitosis, and differentiation of tissue-residing stem cells (Zhou et al., 2009). Indeed, it has been demonstrated that hematopoietic CD34(+) stem cells are able to down-regulate the pro-inflammatory TNF-α, IFN-γ, and IL-1, as well as to up-regulate the anti-inflammatory cytokine IL-10 (Greish et al., 2012) (Figure 1).

## Challenges in the Application of HSCs to ASDs

Although HSCs are attractive candidates for the restoration of ASD-related immune-mediated pathologies, several concerns must be addressed to adequately understand their proper application. As mentioned previously, the BBB may not be properly managing its immunological regulatory functions, and as such it may allow abnormal trafficking of stem cells into the CNS with resultant unintended consequences. Equally, HSCs may offer desirable reparative functions to the BBB.

The reported structural and functional abnormalities of ASD minicolumns present a daunting challenge to HSCs therapeutics. However, progress with animal models should allow for adequate testing of the amenability of minicolumnopathies to HSC interventions.

In a recent review of the bidirectional nature of stem cells to both repair and extinguish inflammatory processes, or in contradistinction, to contribute to the maintenance of the inflammatory state, the authors point out the importance of proper cytokine signaling as deterministic of the end-effect of stem cell responses (Shi et al., 2012). Others have expressed equal concern over stem cell enhancement of inflammatory conditions (Blanchet and McNagny, 2009; Siniscalco et al., 2011).

Thus, one feature of any effective HSC therapeutic application must be consideration for proper cytokine signaling. In particular, anti-inflammatory medications and potentially even the anti-inflammatory effects of biological therapies, e.g., curcumin or luteolin, may interfere with stem cell targeting and allow them to exacerbate the existing inflammatory state. Again, the hope is that animal modeling will assist in directing therapeutic application.

In a similar way, the inflammatory bowel disease associated with autism, may respond in a comparably favorable way to HSCs as reported in the ASTIC trial. But once again caution must be inserted as up-regulation of GMPs were noted in the autism population studied by Walker et al. (2013). A reasonable concern would be the methodologies to prevent recruitment of HSCs to this inflammatory GMP population.

Further complicating our application of HSCs to ASDs, relates to the unresolved, underlying cause for the chronic inflammation. It is reasonable to consider a chronic infectious agent(s) as the maintainer of the immune dysregulations observed. Potentially, the events leading to the inflammation may merely represent the long-term consequences of an ill-timed exposure during gestation or early postnatal life. The latter, would represent a more desirable scenario for HSC therapeutics, while the former presents substantial challenges. This also raises the issue of autologous or allogeneic source material for the HSCs. While using autologous HSCs preclude concerns over graft-versus-host reactions, these stem cells may be programed by the latent infection is undesirable ways. The severe risk of graft-versus-host disease (GVHD), the toxicity of ablative conditioning, and the need for close donor-recipient matching would need to be addressed if allogeneic sources of HSCs were used (Leventhal et al., 2013). However, these serious and life-threatening challenges make allogeneic HSCs undesirable in ASDs.

## Summary

While HSCs are populations of multipotent stem cells that have been identified as promising potential candidates for treating a broad range of conditions; their basic biology remains inadequately characterized to answer all of the questions raised by their use. Despite these concerns, HSCs, based on an internet review of the stem cell providers offering services to patients, are already in use for ASDs. As such, a better understanding of the HSC molecular mechanisms, as well as experimental or clinical data, is urgently needed to provide more data to develop proper strategies to improve the use of these cells in therapy in ASD in a large scale (Kavanagh and Kalia, 2011).

## Conflict of Interest Statement

The authors declare that the research was conducted in the absence of any commercial or financial relationships that could be construed as a potential conflict of interest.
